# Effect of Underload Cycles on Oxide-Induced Crack Closure Development in Cr-Mo Low-Alloy Steel

**DOI:** 10.3390/ma14102530

**Published:** 2021-05-13

**Authors:** Pavel Pokorný, Tomáš Vojtek, Michal Jambor, Luboš Náhlík, Pavel Hutař

**Affiliations:** 1 Institute of Physics of Materials, Czech Academy of Sciences, Žižkova 22, 616 00 Brno, Czech Republic; pokorny@ipm.cz (P.P.); jambor@ipm.cz (M.J.); nahlik@ipm.cz (L.N.); hutar@ipm.cz (P.H.); 2 Central European Institute of Technology (CEITEC), Brno University of Technology, Purkyňova 123, 612 00 Brno, Czech Republic

**Keywords:** underload cycles, crack closure, threshold, fatigue crack growth, oxidation, EA4T steel

## Abstract

Underload cycles with small load amplitudes below the fatigue crack growth threshold are dominantly considered as insignificant cycles without any influence on fatigue lifespan of engineering structural components. However, this paper shows that in some cases these underload cycles can retard the consequent crack propagation quite significantly. This phenomenon is a consequence of oxide-induced crack closure development during cyclic loading below the threshold. The experimentally described effect of fatigue crack growth retardation was supported by measurement of the width and the thickness of the oxide debris layer using the EDS technique and localized FIB cuts, respectively. Both the retardation effect and the amount of oxide debris were larger for higher number and larger amplitudes of the applied underload cycles. Crack closure measurement revealed a gradual increase of the closure level during underload cycling. Specimens tested in low air humidity, as well as specimens left with the crack open for the same time as that needed for application of the underload cycles, revealed no retardation effect. The results can improve our understanding of environmental effects on fatigue crack propagation and understanding the differences between the results of laboratory testing and the fatigue lives of components in service.

## 1. Introduction

Fatigue crack propagation is an important stage of the fatigue failure of engineering components, especially in applications where the damage-tolerance design is employed. In these applications, components are regularly inspected for the presence of fatigue cracks using non-destructive techniques, which brings about the need for a high precision of predictions of residual fatigue life (RFL). The near-threshold regime is the most significant area to study, since the majority of RFLs are associated with the beginning of crack propagation, where the stress intensity factors are small. In addition, the majority of loading cycles in the spectra of variable amplitude loading usually has small amplitudes. Moreover, this area is characterized by many unresolved theoretical questions. In current research, it is desirable to improve our understanding of mechanisms of resistance to fatigue crack growth (FCG) in the near-threshold regime, where the crack closure effects represent a significant part of the resistance, and they are very difficult to predict due to a lack of quantitative models. Crack closure effects are also responsible for the generally large scatter of reported threshold values for particular metallic materials.

**Figure 1:**
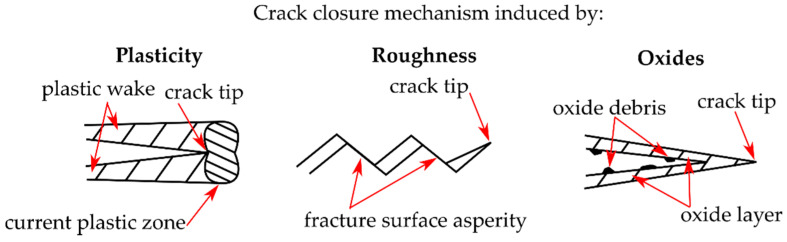
Mechanisms of crack closure due to plastic deformation in the crack wake, fracture surfaces roughness and oxide debris produced on fracture surfaces.

The crack closure phenomenon was firstly described in [1] and is a crack tip shielding mechanism [2] diminishing the crack driving force. Due to premature contact of fracture surfaces, the cyclic plastic zone is reduced and, therefore, crack propagation is slower than in the case of the absence of crack closure effects (such as at high load ratios). The most commonly considered mechanisms of crack closure are due to plastically deformed materials in the crack wake, roughness of fracture surfaces, and the presence of enhanced oxide debris at fracture surfaces, as illustrated schematically in Figure 1. More details about crack closure mechanism can be found in the literature, e.g., [3,4,5]. While plasticity-induced crack closure (PICC) is present in both the near-threshold and the Paris regime, roughness-induced crack closure (RICC) the oxide-induced crack closure (OICC) are significant only in the near-threshold regime owing to small cyclic crack opening displacements.

The effective (closure-free) threshold in steels is usually about 3 MPam^1/2^, while the measured thresholds in terms of *K*_max_ are often two or three times larger. The level of plasticity-induced crack closure (PICC) should result in no more than 1/3 of *K*_max_ in most situations, which means that there is a gap of about 2 to 5 MPam^1/2^ needed to account for RICC and OICC. These two components are difficult to quantify, both experimentally and theoretically. The dependence of the threshold on grain size has been traditionally attributed to the role of RICC. However, it was not clear until recently, what are the proportions between RICC, OICC and the whole threshold value. Experiments in dry air [6] provided one of the first hints on this matter, where the differences between thresholds in dry air and humid air were found to be significant. For railway axle steel EA4T, they were 4 MPam^1/2^ and 7 MPam^1/2^, respectively, in terms of *K*_max_ for the load ratios *R* = 0.1 and *R* = −1. Thus, the role of OICC was much more significant than the role of RICC in this material. There are experimental indications that such a high significance of OICC for threshold values is also valid for other corroding steels [7] and that there is no physical reason to think the otherwise. Thus, the effect of OICC is a good candidate for an explanation of the differences in threshold values measured under various influencing factors. One of these influences is the load shedding technique, often applied to measure thresholds.

**Figure 2:**
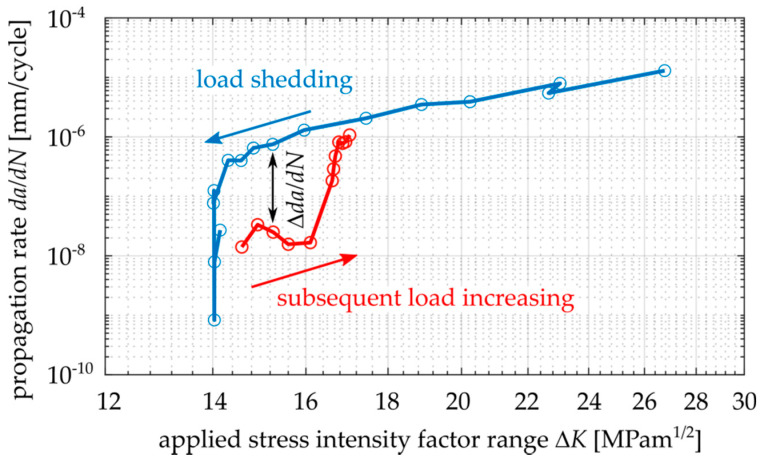
Fatigue crack retardation after load shedding procedure in *da/dN-K_max_* curve, data experimentally obtained for Cr-Mo low-alloy steel, *R* = −1.

Different fatigue crack propagation rates in the near-threshold area were measured for EA4T steel during load shedding and during subsequent loading with an increasing Δ*K*, as shown in Figure 2. The low crack growth rates during increasing Δ*K* can be explained by the gradual development of an oxide debris layer at fracture surfaces during the load shedding procedure and, consequently, a larger oxide-induced crack closure [6,8,9]. A similar behavior of oxide layer development can be expected for cyclic loading below the threshold.

A majority of laboratory experiments have generally been conducted under constant amplitude or quasi-constant amplitude loading, where only slow gradual changes of Δ*K* are applied. However, engineering components in operation are typically subjected to variable amplitude loading (VAL). The load-interaction effects influence RFL in terms of both retardation and acceleration of the crack. The VAL spectra usually have such character that the lowest amplitudes have the highest number of cycles; therefore, not only are the majority of damaging cycles in the near-threshold area, but there is also a large number of non-damaging cycles just below the threshold. The available models of crack propagation under VAL (e.g., [10,11]) include load-interaction effects based on PICC or on the development of residual near-tip stresses. The number of studies on the retardation effect due to OICC is very limited. Only very few publications can be found in the literature on the topic of the effects of underload cycles [12,13,14].

**Figure 3:**
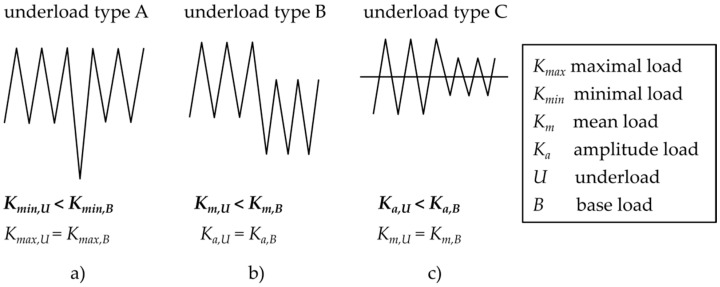
The three types of underloads considered in literature: (**a**) a single underload with the shift of *K_min_*, (**b**) underloads with reduced mean load, and (**c**) underloads with reduced load amplitude.

Referring to the above-mentioned facts, studying the influence of underload cycles is important for structural components in application, as well as for a deeper understanding of fatigue crack propagation mechanisms. In the literature many papers can be found on the topic of the effects of underloads. However, a majority of them, e.g., [15,16,17,18], are related to intense compressive overloads or a reduced mean load (see the underloads of the type A and B in Figure 3a,b). In other words, “underload” means a significant reduction (shift to negative values) of the minimal load. The underload cycles in this work are studied in terms of a reduction of amplitude, while the load ratio is kept constant (*R* = −1). It means that both maximal and minimal values during the loading cycle are shifted closer to the mean load (see the type C underload in Figure 3c). Moreover, the underload cycles considered in this paper are below the threshold of crack propagation, which means that these cycles are non-damaging and are usually considered to have no impact on RFL.

**Figure 4:**
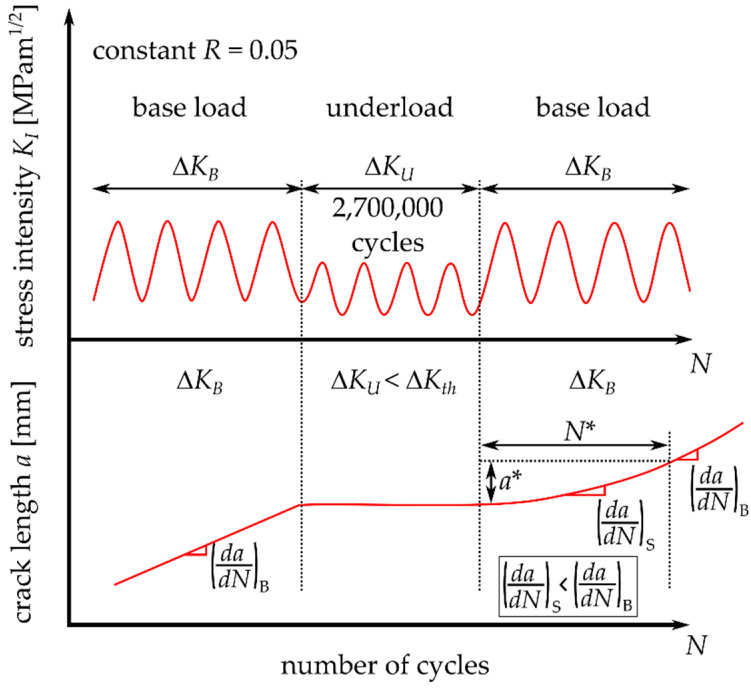
A schematic illustration of the loading sequence used by Suresh and Ritchie in [14]. Adapted with permission from ref. [14]. 1981 Elsevier.

In [12], it was reported that many of the cycles below threshold contributed to crack growth retardation, when the base load was applied after the underloads. More details about this topic for railway axle steels EA4T and EA1N, tested at load ratios *R* = 0.1 and −0.5, are published in [13]. A comprehensive paper by Suresh and Ritchie [14] presented the effects of underloads (type C in Figure 3c) in 2.25Cr-1Mo pressure vessel steel at an ambient temperature and 30% relative humidity. A block of 2,700,000 underload cycles was inserted between base load cycles and the role of the underload cycles on subsequent crack propagation rate was examined (see Figure 4). At loading below the threshold, the crack does not propagate; however, due to cyclic crack opening and closing an oxide debris layer gradually grows.

The presented paper is focused on the role of loading cycles with amplitudes below the threshold on subsequent crack retardation due to the mechanism of OICC in the Cr-Mo low-alloy steel designated EA4T, according to railway standards. The aim is to quantify this retardation effect and the corresponding development of oxide debris layer.

## 2. Experimental Procedure and Methodology

**Figure 5:**
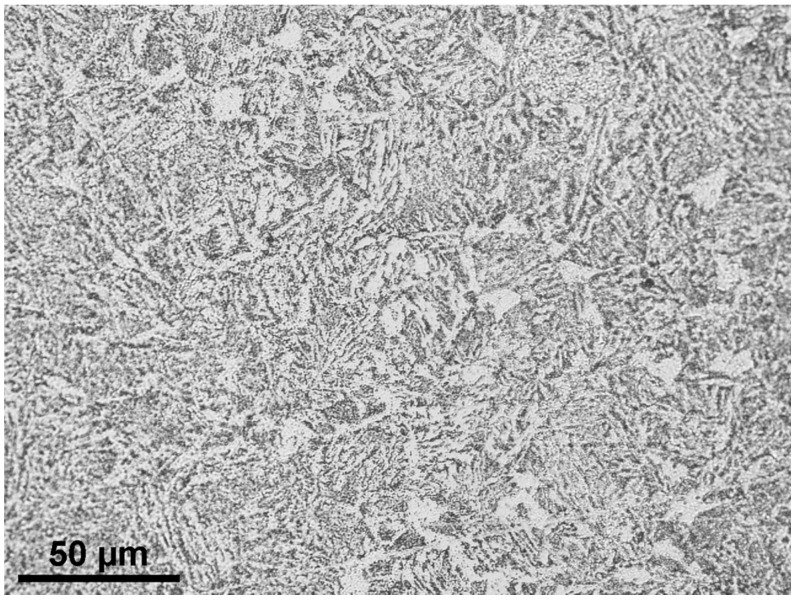
Microstructure of the investigated Cr-Mo low-alloy steel (EA4T steel).

All tests were performed on specimens made of the EA4T steel, which is probably the most commonly used material worldwide for the manufacturing of driving railway axles. Therefore, knowledge of its behavior is important for the safety of train operations. Table 1 shows the chemical composition of this steel. The manufacturing process and heat treatment of the axles lead to a microstructure with a dominant portion of bainite. An optical microscopy image of the etched metallography cut of the material is shown in Figure 5.

**Figure 6:**
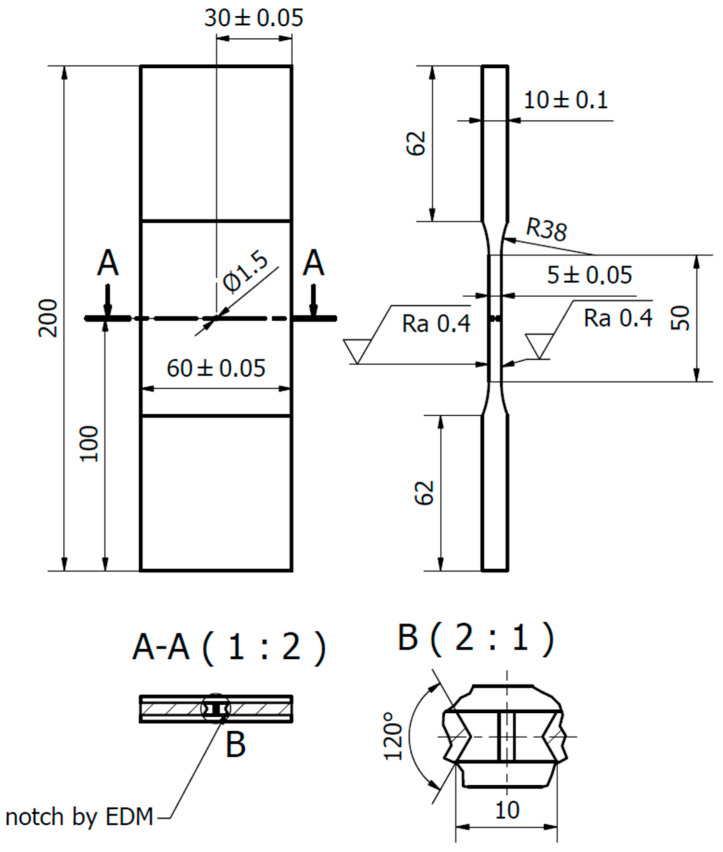
Scheme of the MT specimen used for experimental determination of the underload effects in the EA4T steel. (Unit: mm).

Table 2 presents selected mechanical properties of the investigated EA4T steel. The parameters related to fatigue crack propagation were experimentally determined using the middle-crack tension (MT) specimen (shown in Figure 6). This type of specimen was also used in experimental investigations of underloads effects in this paper. Note that the specimens were manufactured from the surface part of a real axle in order to more realistically capture the state of the material, see details in [20].

**Figure 7:**
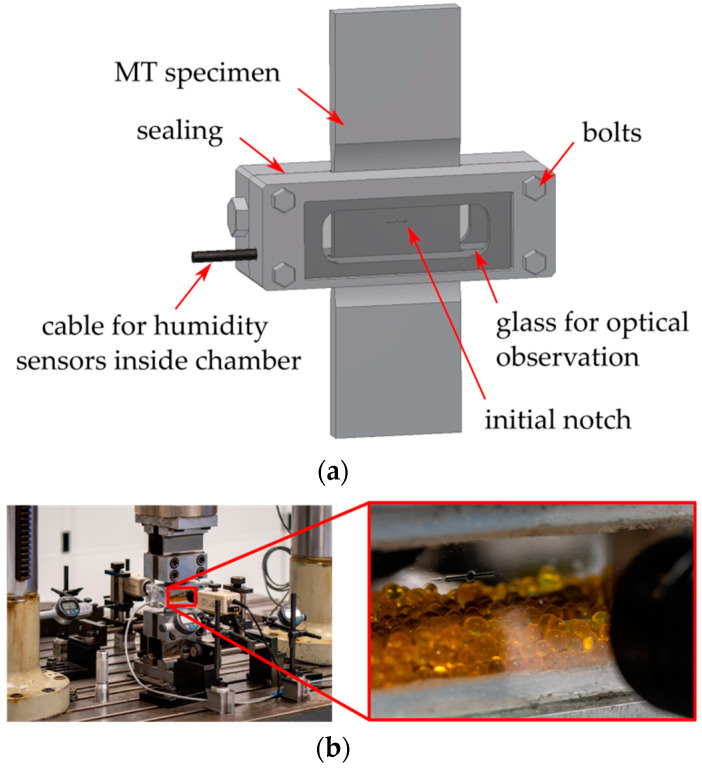
Special test setup for tests in dry air: (**a**) scheme of the sealed chamber and (**b**) image of the experiment showing the crack surrounded by silica gel particles.

All of the used MT specimens included a sharp central notch produced by electro discharge machining (EDM). A chevron notch was used in order to initiate a symmetrical fatigue crack, (detail B in Figure 6). Experimental tests were carried out on a resonant Schenck PVQ machine with a testing frequency of about 50 Hz (depending on specimen stiffness loading amplitude). The majority of experimental tests were carried out in laboratory air with controlled temperature and humidity. The temperature was set to 23 °C and the absolute humidity was set to 10 g/m^3^ (relative humidity of ca. 50%). Some of the tests were carried out using the originally designed chamber for tests in very dry air, see Figure 7. This chamber surrounds the central part of the MT specimen and it is filled with silica gel particles to eliminate air humidity (relative humidity is below 15%). Note that such a low humidity significantly retards the development of oxide debris during crack propagation [6,21].

The crack lengths and crack increments were measured optically using two digital Basler acA2040-55um cameras (with the CMOS sensor, resolution of 3.2 Mpx, frame rate 55 fps) and Azure-2514MM objectives (with the format 2/3″, manual iris F1.4–F32, focal length 25 mm). The cameras were fit to movable tables and were focused on the fatigue crack tip. The positions of the cameras were measured using Mitutoyo ID-C125XB universal digital indicators (with the resolution of 0.001 mm and the accuracy of 0.003 mm).

Each test was performed at a load ratio *R* = −1. Fatigue cracks were initiated from the machined notch with the length of 10 mm (see Figure 6) via cyclic loading at an amplitude of 23 kN. When a fatigue crack measuring 1 mm in length was initiated on both sides of the specimen, the procedure of load reduction by approx. 5% per crack increment of 0.1 mm was stared. The load shedding procedure continued until the crack growth rate reached 2 × 10^−6^ mm/cycle, which was selected as the growth rate where the production of oxide debris is not very intense yet, but the load is small enough to have easily detectable retardation effect after the enhanced oxide layer is developed during the underload cycles.

**Figure 8:**
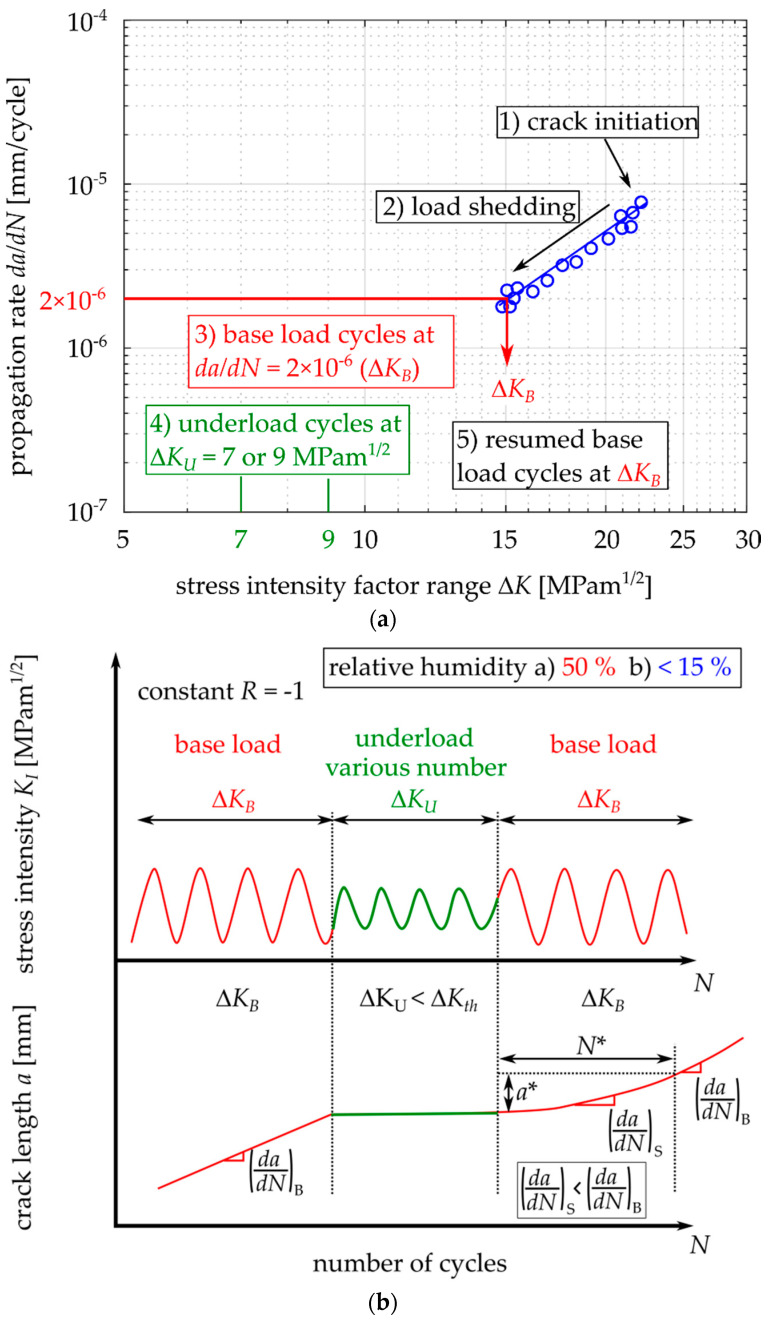
Scheme of the conducted experimental procedure. (**a**) Procedure steps in terms of the crack growth rate diagram; (**b**) Loading sequences. Unlike in Figure 4, various numbers of underload cycles were applied and the humidity conditions were varied.

Next, a certain number of underload cycles was applied, which ranged from 0 up to 30,000,000 cycles. After that, cyclic loading at the same amplitude as before the underload cycles was started (further referred to as the “base load cycling”), see Figure 8. During that loading, the number of influenced load cycles was investigated. It was assumed that the amount of influenced load cycles N* corresponds to the number of cycles until the crack growth rate reaches again the base-load rate (*da/dN*)_B_ = 2 × 10^−6^ mm/cycle, see Figure 8. The crack extension during this period was denoted as the influenced zone, a*.

**Figure 9:**
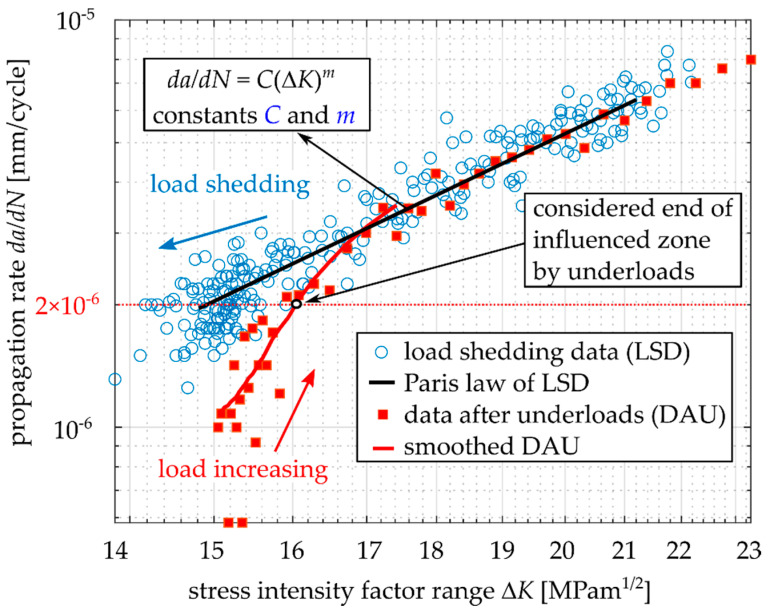
Demonstration of different crack propagation rates before and after application of underload cycles.

Figure 9 shows a typical difference between the data obtained during load shedding (before application of the underload cycles) and the data obtained after the underload cycles. Note that this behavior is very similar to the behavior depicted in Figure 2, where, after the load shedding, no underloads were applied. However, the underload cycles result in a more pronounced effect of crack retardation.

**Figure 10:**
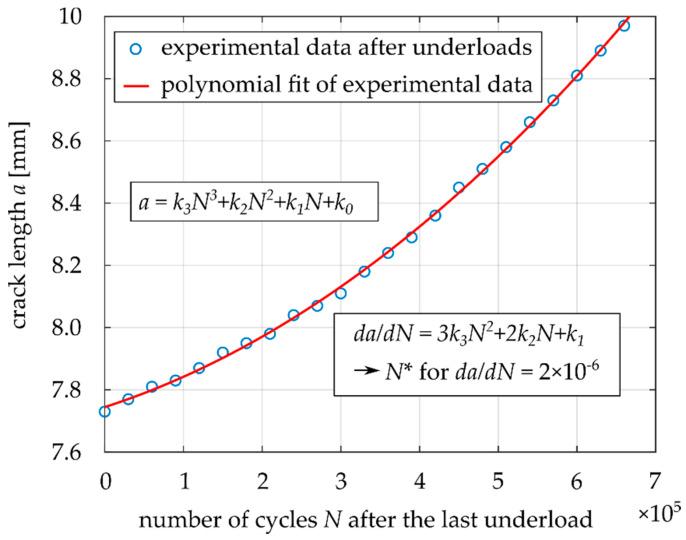
Determination of the number of influenced base load cycles after application of the underload cycles based on the fitting equation.

In order to mathematically express the number of influenced base load cycles after the underload cycles N* (see Figure 8), the *a-N* data after the underload cycles were plotted and fitted by a polynomial function of the 3rd order:

(1) $$a=k_{3}N^{3}+k_{2}N^{2}+k_{1}N+k_{0}$$

where *k_0_*, *k_1_*, *k_2_* and *k_3_* are the polynomial constants, *a* is the crack length and *N* is the number of cycles after the last underload cycle. The typical evolution of the crack length *a* with the number of applied base load cycles *N* is shown in Figure 10.

Estimation of the number of influenced base load cycles N* after application of the underload cycles was done using the derivative of Equation (1):

(2) $$\frac{da}{dN}=3k_{3}N^{2}+2k_{2}N+k_{1}$$

The crack propagation rate before application of the underloads was 2 × 10^−6^ mm/cycle. When this *da/dN* rate is substituted into Equation (2), N* can be expressed as follows.

(3) $$\frac{da}{dN}=2\times 10^{-6}=3k_{3}N^{*2}+2k_{2}N^{*}+k_{1}\;\to N^{*}=\frac{-2k_{2}+\sqrt{{\left(2k_{2}\right)}^{2}-4\times 3k_{3}.\left(k_{1}-2\times 10^{-6}\right)}}{2\times 3k_{3}}$$

When the number of influenced load cycles after the underload cycles is known, the influenced zone a* (see Figure 8) can be estimated by substitution of N* into Equation (1) with a subtraction of the crack length after the underload cycles *a_0_*:

(4) $$a^{*}=k_{3}N^{*3}+k_{2}N^{*2}+k_{1}N^{*}+k_{0}-a_{0}$$

where a* is the influenced zone size, N* is the number of influenced base load cycles, see Figure 8, and *a_0_* is the crack length before, during and after the underload cycles (the crack does not propagate). Equations (3) and (4) were used for calculation of the data summarized in Table 3.

## 3. Results

### 3.1. Fatigue Crack Propagation Rates after Application of Underloads

Table 3 shows the results obtained for 18 MT specimens. In the case of Specimen 1, 3,000,000 underload cycles were applied at a chosen underload level Δ*K_U_* = 7 MPam^1/2^ (*R* = −1). Application of this number of cycles took ca. 17 h. Subsequently, the base load amplitude was reapplied and the crack growth rate increased to *da/dN* = 2 × 10^−6^ mm/cycle after N* = 375,410 load cycles, which corresponded to the influenced zone a* = 0.54 mm. In the case of Specimen 2, the procedure was the same, only the level of underload cycles was Δ*K_U_* = 9 MPam^1/2^. In this case, there were 711,250 influenced load cycles with an influenced zone of 1.02 mm. The difference can be explained by a higher compressive stress at the minimum load during the cycle at *R* = −1. Additionally, a larger crack opening displacement (COD) in the case of loading Δ*K_U_* = 9 MPam^1/2^ is available to accommodate more oxide debris, compared to the COD at Δ*K_U_* = 7 MPam^1/2^, allowing a higher level of OICC.

Instead of the underload cycles, Specimen 3 was loaded only by a static load at *K_max_* = 4.5 MPam^1/2^ (corresponding to Δ*K_U_* = 9 MPam^1/2^ at *R* = −1) for 17 h. This aimed to reveal whether the retardation effect is related to the underload cycles or the time that passed without any damaging load cycles. The results showed that even though the crack is opened and accessible for diffusion of oxygen and water vapor, no crack retardation under the subsequent base load occurred (see Table 3). In the case of Specimen 4, the procedure was the same as for Specimen 3, but the load for 17 h was kept at zero. As was expected, Specimen 4 did not reveal any retardation effect either. In the case of Specimen 7, no underloads were applied after load shedding and the base load was immediately restarted. This experiment, again, did not lead reveal any significant retardation effect.

Specimens 16–18 were used for experiments in reduced air humidity, see Figure 7. The low humidity air (relative humidity below 15%) led to an insignificant effect of the underload cycles in comparison with the previous experiments in air with a relative humidity of 50%. This supported the idea that the OICC mechanism was responsible for the observed retardation after underloads. Note that the low humidity air leads to a significantly lower threshold of about Δ*K_th_* = 8.8 MPam^1/2^, see [21]. This fact resulted in failure of Specimen 17, where the “underload” cycles at Δ*K_U_* = 9 MPam^1/2^ were not really non-damaging. Interestingly, Specimen 16 was also loaded by Δ*K_U_* = 9 MPam^1/2^ but the crack did not grow during 3,000,000 underload cycles. This showed that the applied Δ*K_U_* was really very close to the low-humidity threshold.

**Figure 11:**
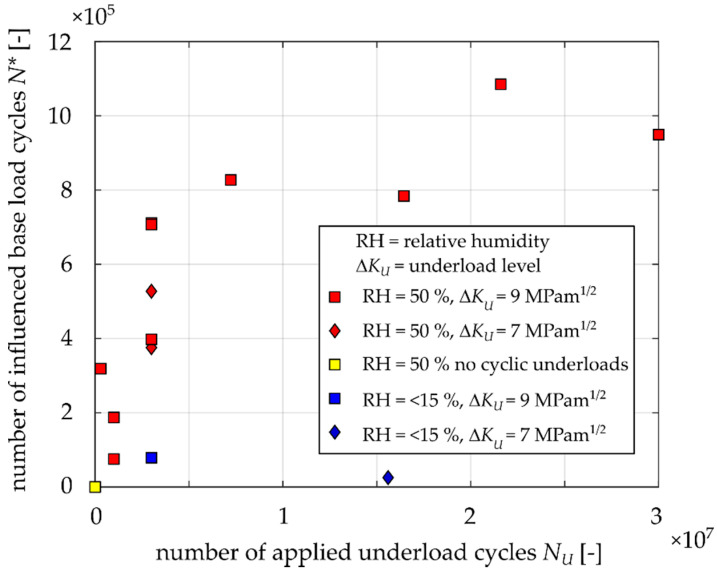
Experimentally obtained numbers of influenced load cycles N* after underloads.

**Figure 12:**
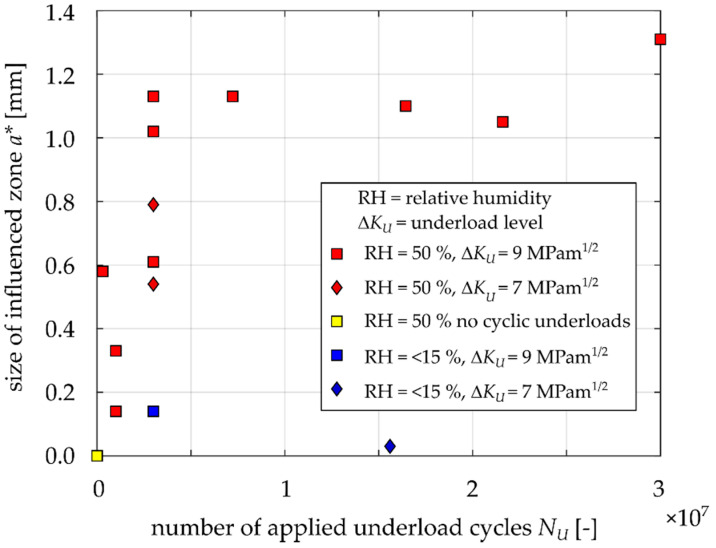
Experimentally obtained influenced zones a* after underloads.

For a better presentation of the obtained results, Figure 11 and Figure 12 show the dependences of N* (number of influenced base load cycles) and a* (influenced zone size) on the number of applied underload cycles *N*_u_. The shapes of both diagrams are very similar. There is a relatively large sensitivity up to ca. 500,000 underload cycles and the dependence is quite flat for higher numbers of underloads. However, there was no clear saturation, even after 10^7^ underload cycles, especially in the case of N* (Figure 12).

In order to have even better expression of the effect of underloads, the numbers of base load cycles needed for crack elongation by 0.5 mm and 1.0 mm were determined for the tested specimens.

The value *N_cal_* represents the number of cycles necessary for the crack extension by 0.5 mm or 1.0 mm without the effect of underloads. The Paris–Erdogan law, with constants given by the load-shedding measurement, was used (see Figure 9). The value *N_exp_* represents the experimentally obtained number of base load cycles needed for crack extension by 0.5 mm or 1.0 mm after application of underload cycles. The relative retardation was calculated according to Equation (5).

(5) $$retardation=\frac{N_{exp}-N_{cal}}{N_{cal}}\cdot 100\;\left[\%\right]$$

Table 4 summarizes the obtained values for a crack extension of 0.5 mm, while Table 5 summarizes the values for a crack extension of 1.0 mm. One can see that, for a relative humidity of 50%, application of 21.6 or 30 million underload cycles leads to more than 100% retardation in comparison with the case of no underload cycles. For dry air (relative humidity below 15%), the effect of underload cycles almost completely vanished. The retardation effect also vanished when no underload cycles were applied (see Specimens 3, 4 and 7 in Table 4). Note that the retardation values of −2% to 2% should be considered more or less as zero due uncertainty in the data measurement.

### 3.2. Width of the Enhanced Oxide Debris Area

After the final fracture of the specimens, it was visible even by the naked eye, that some areas were significantly covered by oxide debris. To characterize the oxide layers, a Tescan Lyra 3 scanning electron microscope (SEM) equipped with a focused ion beam (FIB) column and Oxford Ultimax 100 detector for energy-dispersive X-ray spectroscopy (EDS) was used. The fracture surfaces were analyzed via EDS mapping to reveal the distribution of oxygen within the examined region. Parameters, such as accelerating voltage, beam current, working distance, pixel dwell time, etc., were kept unchanged for all tested specimens to ensure the comparability of the obtained results.

**Figure 13:**
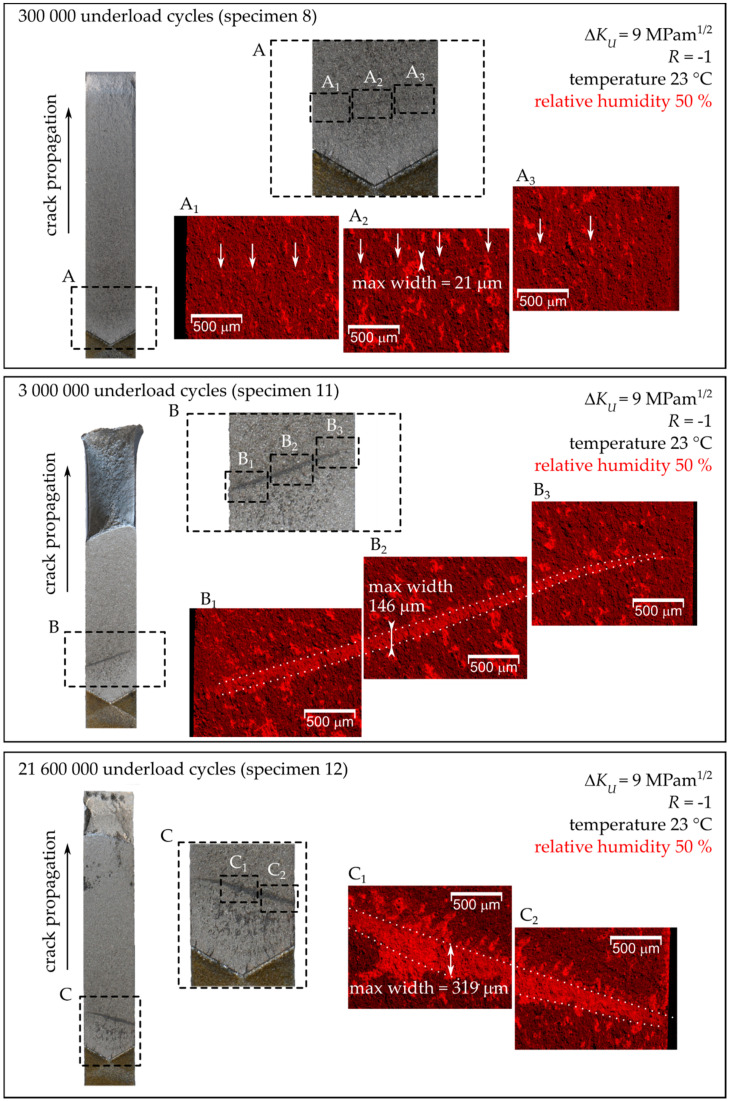
Fracture surfaces of Specimens 8, 11 and 12 tested in 50% relative humidity air (see also Table 3). On the left-hand side, the fracture surface of one quarter of the MT specimen with a detail is shown. The right-hand side shows the EDS maps of distribution of oxide particles.

**Figure 14:**
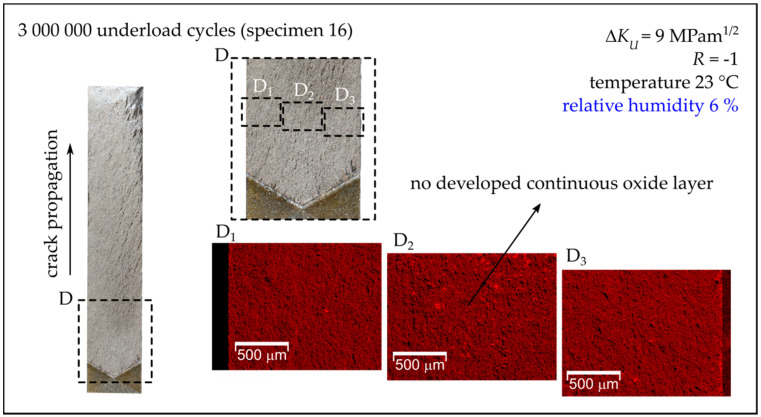
Fracture surfaces of Specimens 16 tested in 6% relative humidity air (see also Table 3). On the left-hand side, the fracture surface of one quarter of the MT specimen with a detail is shown. The right-hand side shows the EDS maps of distribution of oxide particles.

Figure 13 shows the fracture surfaces of chosen MT specimens, which were subjected to a block of underload cycles in 50% humidity air. The bright areas in the EDS maps correspond to the formed oxide layers/particles. The largest amount of oxide debris was in the central part of the specimen, while the oxide layers tended to disappear close to the free surfaces, see Figure 13. The maximal width of the oxide layer in the direction perpendicular to the crack front (not to be confused with thickness) was then measured in the maps for all examined specimens. In the case of specimens subjected to low humidity air (relative humidity below 15%), no continuous oxide layer was found, as can be seen for the representative specimen (Specimen 16) presented in Figure 14.

**Figure 15:**
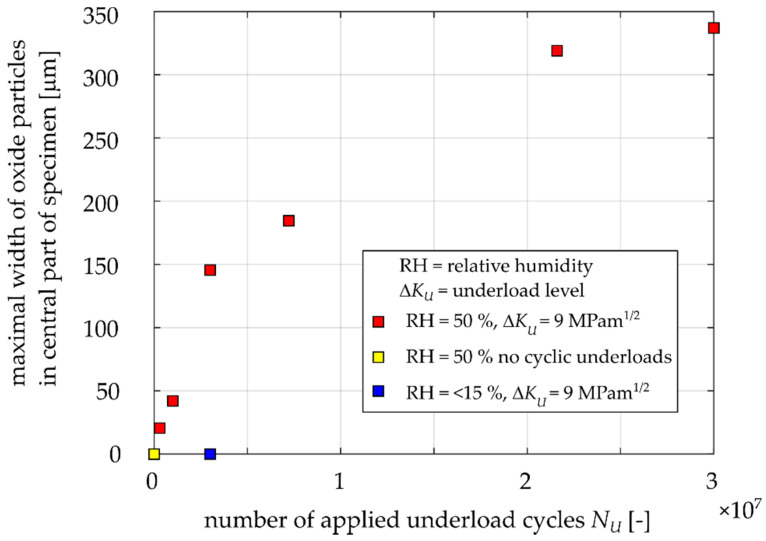
Determined maximal widths of oxide layers for the Specimens 3, 4, 7, 8, 10, 11, 12, 14, 15 and 16 based on EDS maps measurements.

Figure 15 shows the measured widths of the area of oxide debris and the dependence on the applied number of underload cycles. The diagram reveals a clear increasing trend of the oxide layer width with an increasing number of applied underload cycles. After 30,000,000 underload cycles in 50% relative humidity air, the maximal width of the oxide layer was 337 μm. It could be expected from the obtained trend that this value is not a saturated one and that it is likely that this width would further increase with even higher numbers of underload cycles.

For the case of a specimen loaded by a static load of *K_max_* = 4.5 MPam^1/2^ instead of the underloads (a possible situation corresponding to the downtime of a train), no continuous oxide layers were detected. Observation of Specimen 16, tested in dry air (relative humidity 6%), did not reveal any oxide layer even after 3,000,000 underload cycles (see the blue point in Figure 15), while the specimen tested in 50% relative humidity air under an equal number of underload cycles exhibited the maximal oxide layer width of 146 μm.

### 3.3. Thickness of the Oxide Debris Layer

**Figure 16:**
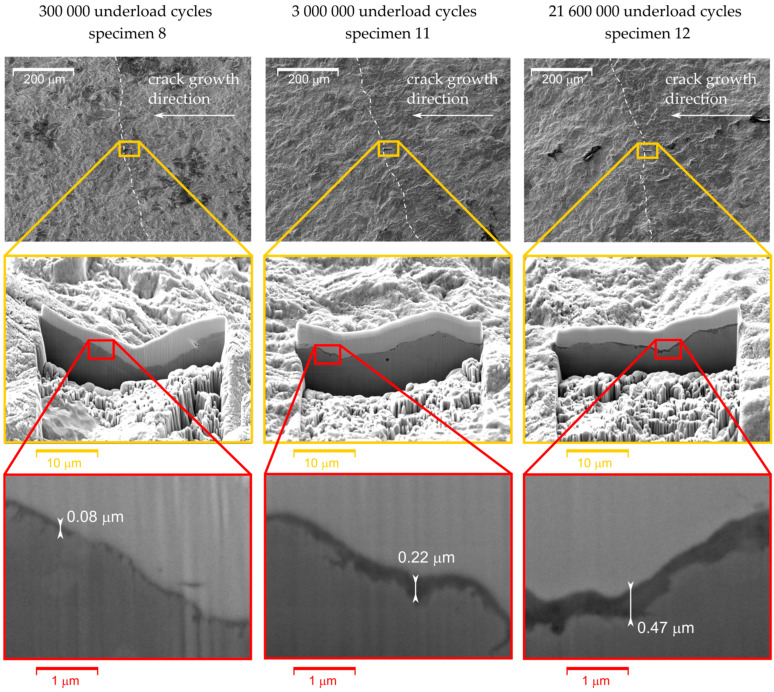
Focused ion beam (FIB) cuts of the selected specimens with highlighted thicknesses of the oxide layers. The position of the actual crack front at the time of application of the underload cycles is marked by the white dash-dotted curve in the top images.

**Figure 17:**
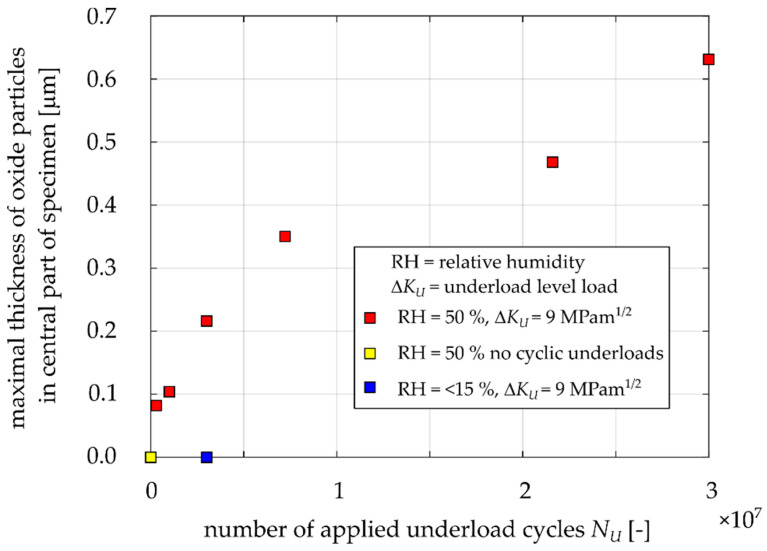
Measured thicknesses of the oxide layers for Specimens 3, 4, 7, 8, 10, 11, 12, 14, 15 and 16 based on the FIB cuts imaging.

Apart from the width of oxidized area of the fracture surfaces, the maximal thickness of this layer was evaluated via milling of the cross section by a focused ion beam (FIB). Note that the oxide layers exhibited a large scatter in the thickness within one specimen; hence, the maximal value was considered as representative for mutual comparison. The FIB cut was made in approximately the same location as where the maximal width of the oxide layer was measured. Proper positioning of the FIB-cuts within the oxide layer was ensured by simultaneous observation of the EDS maps. To protect the oxide layer during milling process, a platinum layer was deposited on the top of the area of interest prior to ion milling procedure. Figure 16 shows the FIB cuts for Specimens 8, 11 and 12. The images reveal that the higher number of applied underload cycles, the thicker the oxide layer generated on fracture surfaces. In the case of Specimen 8, loaded by 300,000 underload cycles, the oxide layer thickness was 0.08 μm, whereas Specimen 12, loaded by more than 21,600,000 underload cycles, exhibited a maximal oxide layer thickness of 0.47 μm. The oxide layer thickness was evaluated for all specimens for which the oxidized zone width was measured (see Figure 15). The results are presented in Figure 17. Through comparison of Figure 15 and Figure 17, it can be seen that the oxide layer thickness is about four orders of magnitude smaller than the oxide width. Nevertheless, the trends of both dependencies are very similar. Figure 17 also confirms that the absence of underload cycles, as well as reduced air humidity, lead to suppression of oxide layer development.

### 3.4. Crack Closure Development during Underload Cycling

**Figure 18:**
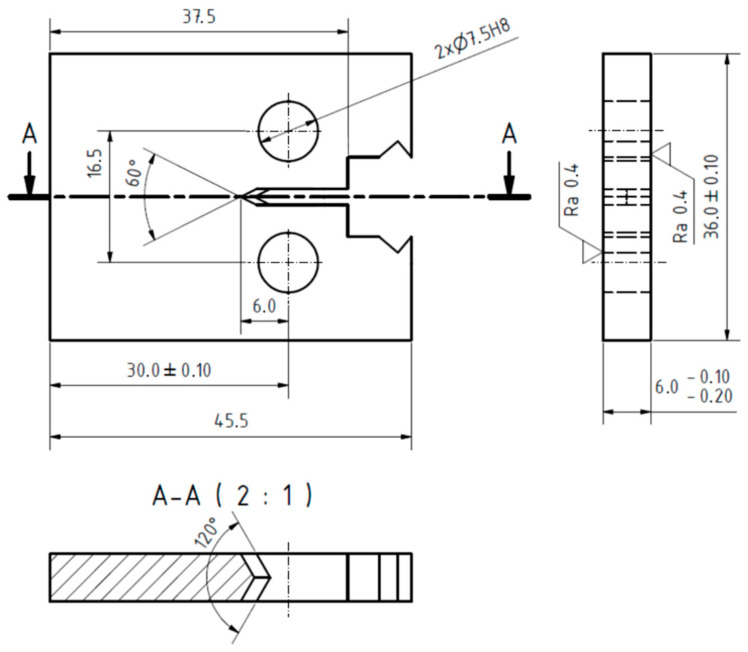
CT specimen used for determination of crack closure behavior. (Unit: mm).

Based on the presented results, it is clear that during the underload cycling the width and thickness of the oxide debris layer gradually increases. Hence, it is expected that oxide-induced crack closure will also increase (see, e.g., [3,6]). Therefore, the level of crack closure was measured in a special experiment. Unfortunately, the MT specimens are not suitable for crack closure measurement during application of many cycles, since the COD gauge cannot be simply used and the strain gauges either provide too weak signals or the period of cycling during which measurement is possible is too short. Therefore, the compact tension (CT) specimen was used to record the evolution of crack closure in dependence on the number of applied underload cycles. The CT specimen with parameters *W* = 30 mm and a thickness of 6 mm, depicted in Figure 18, was tested with an Instron ElectroPuls e3000 machine at a frequency of 60 Hz. Crack closure was determined using the COD gauge (Sandner EXR10-1o) connected to crack mouth of the specimen (also called a CMOD gauge). The compliance change method according to the ASTM standard E647 [22] with an offset of 4% was employed to determine the crack closure level. It should be noted that such determined values correspond to the so called “remote” crack closure data [23,24]. However, such data are sufficient for the purpose of observation of proportional changes of the crack closure level.

The load procedure was the same as in the case of the MT specimen (see Figure 8). When the crack growth rate reached *da/dN* = 2 × 10^−6^ mm/cycle, the underload cycles were launched at *R* = 0.1 and *K_max_* = 4.5 MPam^1/2^, which was the same *K_max_* as in the cases of the MT specimen tests. The tests were running in loop sequence, where one loop consisted of the application of 50,000 underload cycles at 60 Hz and 3 underload cycles at 0.2 Hz. The low frequency ensured error reduction of the measured data by the COD gauge.

**Figure 19:**
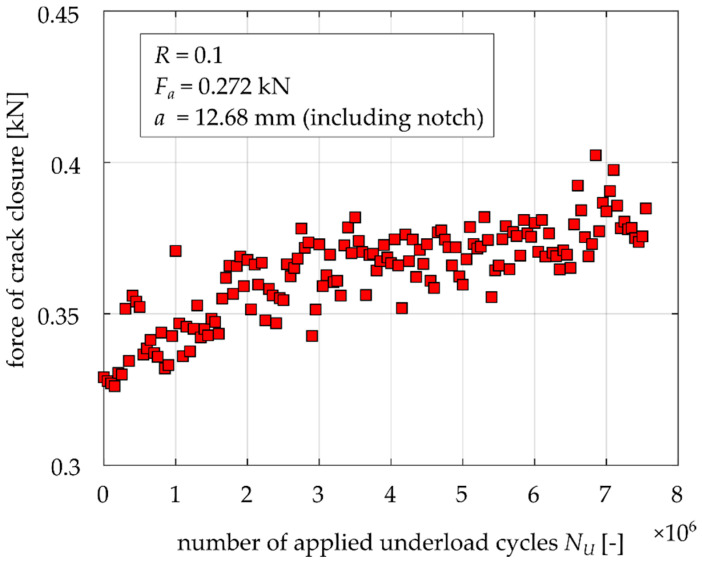
Experimental results of crack closure as function of underload cycles.

Each point in Figure 19 represents the determined crack closure level every 50,000 cycles in the range of 0–7,500,000 underload cycles. It is clear that crack closure is permanently increasing and there is no detectable saturation even after such large amount of underload cycles. This result is in agreement with the data shown in Figure 11 and Figure 12, where no saturation can be deduced either.

## 4. Discussion

### 4.1. Factors Influencing the Experimental Results Obtained by Various Methodologies

In general, crack closure is dominantly caused by the three mechanisms described in the Introduction. The results of the experiments and crack closure measurement showed that OICC is probably a very significant mechanism of crack closure under the investigated loading regimes. On the other hand, the role of PICC can perhaps be neglected during application of the underload cycles, since the crack is not growing and no significant plasticity effect should be expected. The role of RICC during application of the underload cycles could almost be neglected as well, since the crack front stays at the same position. Nevertheless, considering the permanent cycling with fracture surface contact could lead to flattening of the crack flanks, which would decrease the crack closure level. However, Figure 19 shows that there is increase in crack closure during underload cycling, at least for the amount of underload cycles used in the presented experiments. It does not disprove the possibility of change of RICC after application of 10^8^ or 10^9^ cycles, which correspond to the problems in the field of very high cycle fatigue. Therefore, the observed retardation effects can be assigned to the OICC mechanism.

As was pointed out in [14], the oxide layer thickness is proportional to crack opening displacements near the crack tip during the underload cycles. This is also valid for the herein investigated material, EA4T steel (see the shift in data between specimens underloaded at Δ*K_U_* = 9 MPam^1/2^ and Δ*K_U_* = 7 MPam^1/2^ in Figure 11 and Figure 12). Nevertheless, this paper also reveals that, in addition to the underload level, the oxide debris thickness also depends on the number of applied underload cycles and the ambient humidity. It was shown that a higher number of underload cycles leads to greater fatigue crack growth retardation due to the development of oxide layer at fracture surfaces during underload cycles. The development is more pronounced in humid air than in dry one. According to Figure 12, the retardation influenced zone can be longer than 1 mm for 5 million or more underload cycles at load level Δ*K_U_* = 9 MPam^1/2^, *R* = –1, T = 23 °C and 50% relative humidity. Note that the experimentally obtained size of the influenced zone by oxides is not in agreement with the size estimated by the formula published in [13], even though the investigated material was the same (EA4T steel).

The results obtained in this paper can explain some of the confusing data about oxide thickness at the crack propagation threshold of EA4T, published in [21], where a larger oxide layer thickness of 130 nm was obtained for a relative humidity 30% and a lower oxide layer thickness of 90 nm for a relative humidity 50%. The reason is that there is, not only a dependency on humidity but also on the number of “underload cycles”, which are present in the load shedding procedure for the determination of the fatigue propagation threshold. Generally, the specimens are usually subjected to various amounts of “underload cycles” below the threshold at the end of the load shedding procedure before a particular load level is declared as the threshold value.

The results in Table 4 and Table 5 lead to similar conclusions, demonstrating that the selection of the parameter of crack extension of 0.5 mm or 1 mm is not so important for the methodology. It should be noted, however, that for railway axles in operation, a crack increment from 1 mm to 2 mm can cover a significant portion of the RFL.

### 4.2. Significance of the Results for Residual Fatigue Life of Structural Components

Generally, it can be said that an initial crack length of 1 mm or 2 mm is typically considered for estimation of RFL in the frame of the damage tolerance approach. The size of the considered defect (crack) substantially depends on the acceptable risk and the probability of detection of the crack via non-destructive testing methods [25].

**Figure 20:**
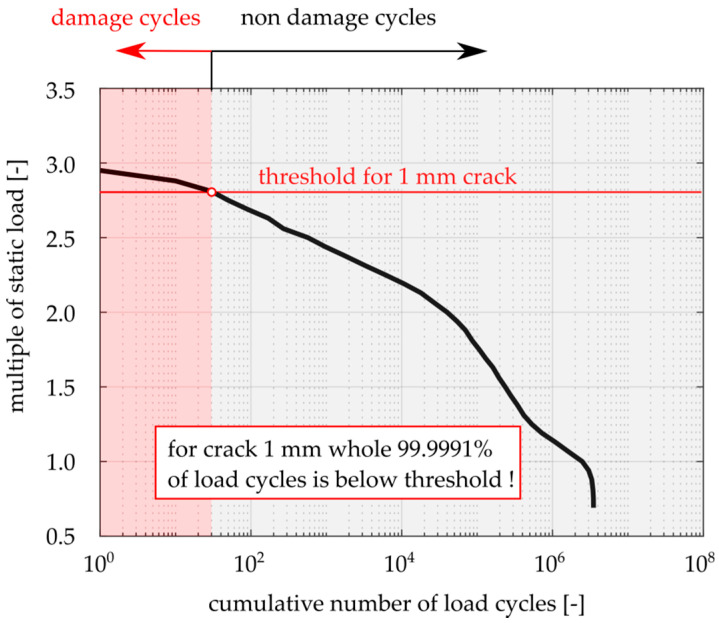
Typical load spectrum corresponding to 10,000 km of train operation.

Variability of the loading amplitude in operation is often described by the so-called load spectra, where the multiple of the static load is on the vertical axis and the cumulative number of cycles is on the horizontal axis. Figure 20 shows a typical example of a load spectrum for railway axles made of EA4T material [26], which corresponds to a block of loads repeated every 10,000 km of train operation. This figure also shows a horizontal line corresponding to the threshold for the material with a 1 mm semi-elliptical surface crack. This line splits the load spectrum into two parts. The first part with the loads above the threshold corresponds to the cycles contributing to crack propagation. The second part with the loads below the threshold, which can be considered as an analogy to the underload cycles considered in this work, corresponds to 99.9991% of the total number of loading cycles in the spectrum.

Therefore, at the start of propagation of the 1 mm fatigue crack, the vast majority of the loading cycles from the load spectrum are non-damaging. After some crack extension (or considering a longer initiation crack), the number of damaging cycles increases, however, the non-damaging cycles are still in the majority, unless the crack is so long that the rest of the fatigue life is already negligible in comparison with the total RFL. Hence, the effect of underload cycles studied in this paper is very relevant to consider in applications.

It is common that the newly designed components are tested in laboratories, considering the so-called omission level method, where small loading amplitudes from the load spectrum are excluded from the experiment in order to save on testing time. It is commonly assumed that these small load amplitudes do not contribute to crack growth behavior. However, in the case of the load spectrum shown in Figure 20, the number of damaging cycles is very small and, moreover, crack propagation under these cycles is expected to be retarded due to the underload cycles. Therefore, the difference between the results of the omission level method and the crack behavior under application of the full load spectrum can be very significant. To conclude, omission of the effect of underload cycles due to OICC in both prediction of RFL and the full-scale tests can lead to excessively conservative results.

## 5. Conclusions

The influence of underload cycles on subsequent crack growth in the near-threshold regime was experimentally investigated for the railway axle steel, EA4T. It was found that the underload cycles, which are below the crack propagation threshold, lead to formation of an oxide debris layer at fracture surfaces, causing an increase in crack closure and retardation of the following crack propagation. The oxide debris layers were measured in terms of thickness and width (direction perpendicular to the crack front). Both the retardation effect and the amount of oxide debris were larger for higher numbers of applied underload cycles, higher amplitudes of the underload cycles and higher levels of air humidity. The results can be summarized as follows:

- Application of three million underload cycles at Δ*K_U_* = 9 MPam^1/2^ (*R* = −1), corresponding to approx. 65% of the threshold of the investigated steel, resulted in retardation of the subsequent crack growth for about 0.6 to 1.1 mm during 4 to 7 × 10^5^ base load cycles. The corresponding enhanced oxide layer had a width of 146 μm and thickness of 0.22 μm.
- Application of 30 million underload cycles at the same load level resulted in retardation of the subsequent crack growth for about 1.3 mm during 1 × 10^6^ base load cycles. The corresponding enhanced oxide layer had a maximal width of 337 μm and maximal thickness of 0.63 μm.
- Application of underload cycles at Δ*K_U_* = 7 MPam^1/2^, corresponding to approx. 50% of the threshold, resulted in thinner oxide layers and shorter influenced crack propagation compared to those of the underloads at Δ*K_U_* = 9 MPam^1/2^ and the same number of cycles. This can be attributed to lower compressive stress in the negative part of the cycle at *R* = −1 or to a smaller crack opening displacement, which leaves less available space in the crack wake for oxide debris development.
- Measurement of the level of crack closure revealed a gradual increase in crack closure during application of the underload cycles.
- The experiments in dry air showed no or negligible effects of the underload cycles on subsequent crack growth and no enhanced oxide debris layer. This confirmed that the oxide-induced crack closure mechanism was responsible for the retardation effect in normal air humidity.
- A verification experiment was done with a static constant load corresponding to *K*_max_ of the underload cycles, where the specimen stayed in the testing machine for the same time as what it takes to apply the underload cycles. There was no detectable retardation of the subsequent crack growth and no enhanced oxide layer developed, which confirmed that the effect is caused by the underload cycles and not by any kinds of gas diffusion dynamics or material creep.
- The significance of the effect of underload cycles in applications was demonstrated on a 1 mm surface crack in a railway axle, where 99.9991% of the loading cycles lie below threshold according to the operational load spectrum. Considering the effect of underload cycles in estimation of residual fatigue life of railway axles can lead to better accuracy.

The results contribute to understanding of mechanisms of resistance to fatigue crack growth in the near-threshold area in terms of oxide-induced crack closure in materials prone to corrosion.

## Figures and Tables

**Table 1:** Chemical composition of EA4T steel, according to [19].

Component	C	Si	Mn	P	S	Cr	Cu	Mo	Ni	V	Fe
min [wt.%]	0.22	0.15	0.50	0.00	0.000	0.90	0.00	0.00	0.00	0.00	rest
max [wt.%]	0.29	0.40	0.80	0.02	0.015	1.20	0.30	0.30	0.30	0.06	

**Table 2:** Mechanical properties of the investigated EA4T steel. Values related to fatigue crack behavior were determined on the middle crack tension specimens (MT) with the width of 60 mm, thickness of 5 mm at 23 °C and 50% relative humidity.

Mechanical Property	Value
Yield stress	*S_y_* = 600 MPa
Cyclic yield stress	*S_y,c_* = 470 MPa
Ultimate tensile strength	*S_U_* = 720 MPa
Young’s modulus	*E* = 204 GPa
Poisson’s ratio	*ν* = 0.3
Threshold value for *R* = −1 (MT specimen)	Δ*K_th_* = 13.8 MPam^1/2^
Threshold value for *R* = 0.8 (MT specimen)	Δ*K_th_* = 2.9 MPam^1/2^
Paris–Erdogan constant *C* for *R* = −1	*C* = 1.53 × 10^−10^ for d*a*/d*N* in [mm/cycle]
Paris–Erdogan constant *m* for *R* = −1	*m* = 3.46
Paris–Erdogan constant *C* for *R* = 0.8	*C* = 1.36 × 10^−8^ for d*a*/d*N* in [mm/cycle]
Paris–Erdogan constant *m* for *R* = 0.8	*m* = 2.72

**Table 3:** Experimentally obtained results of the underload effect on crack propagation behavior: *N_U_*—number of applied underload cycles, *t_U_*—time related to underload cycling or time between the base loading segments, Δ*K_U_* —level of underloads, *RH*—relative humidity, N*—number of influenced load cycles according to Equation (3), a*—influenced zone due to underloads according to Equation (4).

Specimen	*N_U_* [–]	*t_U_* [h]	Δ*K_U_* [MPam^1/2^]	*RH* [%]	N* [–]	a* [mm]
1	3,000,000	17	7	50	375,410	0.54
2	3,000,000	17	9	50	711,250	1.02
3 ^1^	0	17	static load at *Kmax*	50	0	0.00
4 ^2^	0	17	0 (no load)	50	0	0.00
5	3,000,000	17	9	50	706,976	1.13
6	3,000,000	17	7	50	527,365	0.79
7 ^3^	0	0	no underloads	50	0	0.00
8	300,000	2	9	50	318,761	0.58
9	1,000,000	6	9	50	187,009	0.33
10	1,000,000	6	9	50	75,342	0.14
11	3,000,000	17	9	50	397,936	0.61
12	21,600,000	122	9	50	1,084,990	1.05
13	16,440,000	93	9	50	784,028	1.10
14	30,000,000	170	9	50	949,323	1.31
15	7,220,000	41	9	50	827,191	1.13
16	3,000,000	17	9	<15	78,627	0.14
17 ^4^	failed		9	<15	failed	
18	15,600,000	88	7	<15	25,158	0.03

^1^ after reaching *da/dN* = 2 × 10^−6^ mm/cycle, specimen 3 was loaded by a static load of *K_max_* = 4.5 MPam^1/2^ for 17 h (the same time as specimen 1 and 2), ^2^ no loading was applied at all (either underloads or static load) for 17 h, then the base load cycling was resumed, ^3^ in the case of Specimen 7, only the load shedding procedure down to the rate of 2 × 10^−6^ mm/cycle was applied with immediate base load cycling at this level (no loading or pause was applied), ^4^ Specimen 17 failed during the underload cycles due to low humidity that resulted in a lower threshold than Δ*K_U_* = 9 MPam^1/2^ (see [21]) and in subsequent crack propagation.

**Table 4:** Numbers of base load cycles needed for crack increment of 0.5 mm with (*N_exp_*) and without (*N_cal_*) the effect of underload cycles.

Specimen	*N_U_* [–]	*RH* [%]	*N_cal_* [–]	*N_exp_* [–]	Retardation
1	3,000,000	50	234,187	354,545	51%
2	3,000,000	50	256,174	412,941	61%
3	0	50	218,250	237,522	9%
4	0	50	230,871	228,529	−1%
5	3,000,000	50	255,019	350,167	37%
6	3,000,000	50	230,516	376,923	64%
7	0	50	236,181	235,000	−1%
8	300,000	50	238,211	275,069	15%
9	1,000,000	50	220,487	255,732	16%
10	1,000,000	50	213,365	234,512	10%
11	3,000,000	50	234,073	307,286	31%
12	21,600,000	50	243,481	577,167	137%
13	16,440,000	50	226,622	422,500	86%
14	30,000,000	50	212,756	443,360	108%
15	7,220,000	50	233,103	416,842	79%
16	3,000,000	<15	238,956	247,619	4%
18	15,600,000	<15	246,423	245,294	0%

**Table 5:** Numbers of base load cycles needed for crack increment of 1.0 mm with (*N_exp_*) and without (*N_cal_*) the effect of underload cycles.

Specimen	*N_U_* [–]	*RH* [%]	*N_cal_* [–]	*N_exp_* [–]	Retardation
1	3,000,000	50	449,134	568,065	26%
2	3,000,000	50	495,105	703,637	42%
3	0	50	410,749	447,720	9%
4	0	50	433,477	441,892	2%
5	3,000,000	50	487,622	643,033	32%
6	3,000,000	50	442,682	628,571	42%
7	0	50	446,130	457,531	3%
8	300,000	50	454,380	517,891	14%
9	1,000,000	50	416,774	460,091	10%
10	1,000,000	50	401,090	438,100	9%
11	3,000,000	50	448,896	548,273	22%
12	21,600,000	50	473,971	1,048,283	121%
13	16,440,000	50	437,490	741,000	69%
14	30,000,000	50	412,623	795,167	93%
15	7,220,000	50	449,260	720,000	60%
16	3,000,000	<15	455,752	466,667	2%
18	15,600,000	<15	468,201	459,167	−2%

## Data Availability

The data presented in this study are available on request from the corresponding author.
